# Severe mitral valve insufficiency caused by standard surgical aortic valve implantation and its reparation using suture-less prosthesis

**DOI:** 10.1186/s13019-022-01896-6

**Published:** 2022-06-18

**Authors:** Mahmoud Al-Obeidallah, Marian Kohut, Milan Štengl

**Affiliations:** 1 Cardiac surgery department of Faculty Hospital in Pilsen, Charles University in Prague, Alej Svobody 80, 304 60 Pilsen, Czech Republic; 2 Department of Physiology, Faculty of Medicine in Pilsen, Charles University, Alej Svobody 1655/76, 323 00 Pilsen, Czech Republic

**Keywords:** Suture-less prosthesis, Aortic valve replacement, Mitral regurgitation, Aorto-mitral continuity

## Abstract

**Background:**

Aortic valve stenosis is the most frequent cardiac valve pathology in the western world. Surgical aortic valve replacement is the gold standard for the treatment of significant degenerative aortic valve diseases.

**Case presentation:**

This case report highlights an unexpected abnormal iatrogenic shortening of the aorto-mitral continuity and its deformity, during traditional AVR using sutured stented aortic prosthesis as the first choice, which caused significant mitral valve regurgitation. The suture-less prosthesis was a rescue choice to restore the geometry and eliminate the deformation of the aorto-mitral continuity.

**Conclusions:**

Aortic valve replacement using suture-less prosthesis could be a valuable optional choice for lowering the risk of deformation of the aortic annulus and aorto-mitral continuity. It might provide better outcomes in combined procedures.

## Background

Aortic valve stenosis is the most frequent cardiac valve pathology in the western world, with a prevalence of 3% for individuals over the age of 75 years [1]. The incidence of aortic valve stenosis is growing as the reflection of the rapid ageing of the population [2]. Surgical aortic valve replacement is the gold standard for the treatment of significant degenerative aortic valve diseases [3]. During the surgical aortic valve replacement procedure it is important to take into account, among other things, the closely adjacent anatomical structure of the aorto-mitral continuity and the influence of the aortic valve replacement on the functionality of the native mitral valve.

This case report is an overview of a complication of surgical aortic valve replacement procedure using the standard sutured stented valve implantation. A deformity of the aorto-mitral continuity has occurred after the implant, causing severe native mitral valve regurgitation based on the loss of mitral valve leaflets coaptation by newly formed traction force on the base of the anterior mitral valve leaflet. The situation has been solved using the suture-less aortic bio-prosthesis redo-implant option trying to decompress the aorto-mitral continuity and restore the normal function of the native mitral valve.

Another potential advantage of using the suture-less aortic valve prosthesis is a reduction of cardiopulmonary bypass time including reduced cross-clamp time. The use of suture-less aortic valve prosthesis allowed to facilitate minimally invasive as well as complex cardiac surgery procedures while maintaining satisfactory or even improved hemodynamic performance with low incidence of para-valvular leaks [4] over the regular stented surgical bio-prosthesis or TAVI valves. With this case we report another possible advantage using the suture-less valve.

This case report highlights an unexpected abnormal iatrogenic shortening of the aorto-mitral continuity and its deformity, during traditional AVR using sutured stented aortic prosthesis as the first choice, which caused significant mitral valve regurgitation. The suture-less prosthesis was a rescue choice to restore the geometry and eliminate the deformation of the aorto-mitral continuity.

## Case presentation

We present a 57-year-old patient with hemodynamically significant aortic stenosis and normal mitral valve function as reported on echocardiogram before surgery. The patient did not have a preoperative CT scan aortic annulus measurement; we usually rely on preoperative echocardiographic examination, which showed among others, the diameter of the aortic valve annulus about 27 mm.

The patient underwent a surgical aortic valve replacement (SU AVR) by traditional stented aortic prosthesis (Edwards bio-prosthesis of the size 25 mm) implanted using pledgeted U-stitches.

Before the first operation, the patient had both TTE and TEE examination, which showed normal function of the mitral valve. With regard to the standard course of the first operation, perioperative TEE was not performed and consequently, MR was only detected about one week after the operation.

**Fig. 1:**
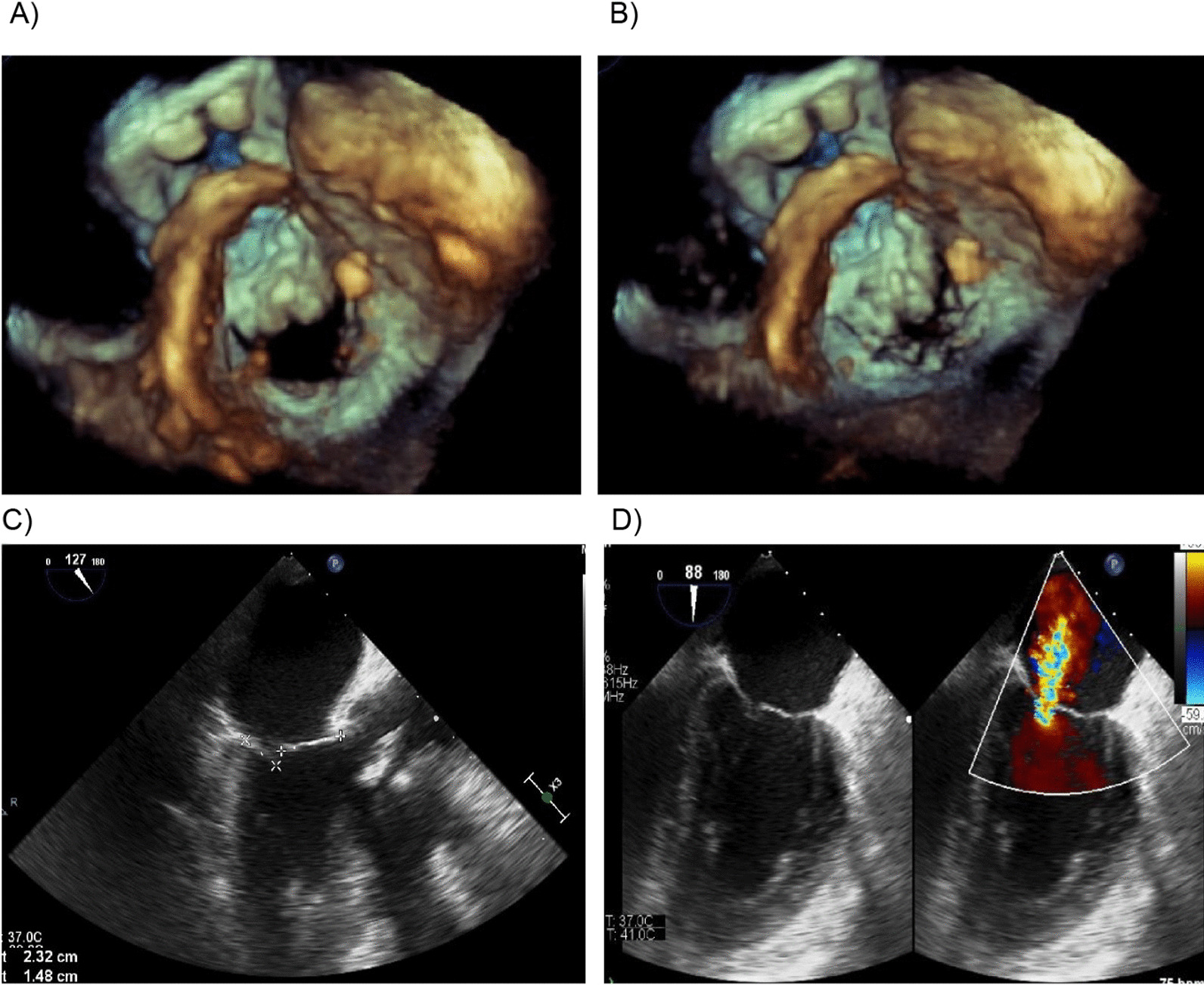
Echocardiogram images after SU AVR using traditional sutured stented aortic prosthesis. **A** Mitral valve orifice—diastolic opening. **B** Mitral valve dysfunction during systolic leaflet closure, loss of coaptation. **C** Slightly restrictive anterior leaflet, impaired apposition and loss of coaptation of the mitral valve. **D** Severe mitral regurgitation showed by color Doppler echocardiography

About a week after SU AVR the control echocardiogram revealed good function of the aortic valve, trace to small intra prosthetic regurgitation with gradient 37/16 mmHg, and severe mitral valve regurgitation which has occurred as a result of slightly restrictive anterior leaflet, impaired apposition and non-coaptation of the mitral valve causing significant eccentric mitral regurgitation, presumably due to the aortic prosthesis implantation (Fig. 1). Furthermore, the patient developed a third-degree atrioventricular block, which required permanent pacemaker implantation.

Redo SU AVR was indicated, the aortic valve prosthesis was explanted and a new bio-prosthesis of the same type Edwards of the size 25 mm was implanted in the second operation with no change in mitral regurgitation. The perioperative echocardiogram showed aortic prosthesis with good function with small intra prosthetic regurgitation, again complicated with significant mitral valve regurgitation.

To correct the severe mitral regurgitation, the mitral valve repair using annuloplasty ring was attempted. Left atriotomy was performed, but the mitral valve was absolutely inaccessible and the surgeon was not able to expose and reach properly the mitral valve. Therefore, the option of mitral valve repair surgery was abandoned and the left atriotomy was closed.

**Fig. 2:**
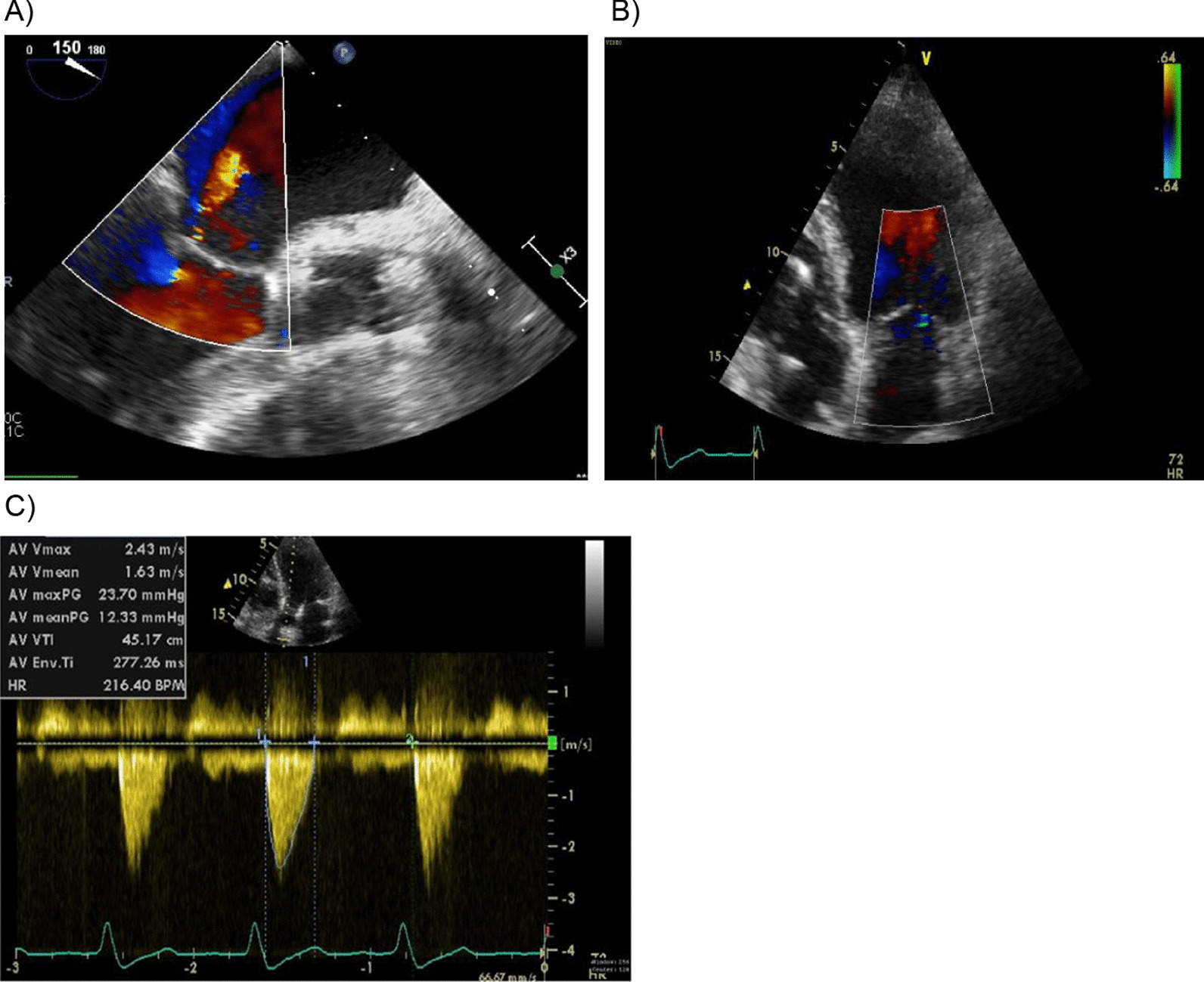
Control Echocardiograms after using suture-less bio-prosthesis (end-results). **A** Perioperative control showing small regurgitation of the mitral valve. **B** Restoration of the mitral valve geometry and function after 1 year. **C** Hemodynamic parameters of suture-less prosthesis after 2 years

Subsequently, as a rescue option, the sutured aortic bioprosthesis was explanted to restore the original geometry of the A-M continuity and the missing aortic valve was replaced with the suture-less aortic bio-prosthesis (Perceval S, LivaNova ®, XL size based on annulus measurement). The perioperative and early postoperative echocardiograms showed good results: small regurgitation on the mitral valve, no paravalvular leaks around the aortic suture-less prosthesis and even lower transvalvular gradients (22/12 mmHg) than the previously used stented aortic prosthesis (Fig. 2A, B). Control echocardiograms after one and two years showed normal function of mitral valve, the suture-less prosthesis with stable function and excellent hemodynamics parameters (transvalvular gradients 22/11 mmHg after one year, 24/12 mmHg after two years) (Fig. 2C), and persistent absence of any paravalvular leaks.

## Discussion

The interest in less invasive cardiac surgery procedures grows steadily in the community of cardiac surgeons. They could allow competing with the non-invasive cardiology procedures and having better long-term results and less patient trauma. The aortic suture-less bio-prosthesis implantation is a technically feasible and safe procedure capable to fulfill this demand. Suture-less aortic valve replacement (AVR) becomes an emerging alternative to standard AVR [5] especially performed with minimally-invasive approach (partial sternotomy/parasternal approach).

Potential advantages include shorter aortic cross-clamp times due to easier and faster implantation during minimally invasive surgery or combined cardiac surgery procedures [6]. Several case series have shown good early clinical and hemodynamic outcomes with the use of suture-less prosthesis [7–9].

In addition, the design of the suture-less Perceval S valve with the flexible stent allows to better preserve physiologic movements and geometry of the aortic root and to avoid the deformation of aorto-mitral continuity. Indeed, there are studies showing that suture-less aortic valves have larger effective orifice area than stented valves [10].

There was definitely a connection between the sutured aortic valve replacement and development of the mitral valve insufficiency. The sutured aortic bio-prosthesis and the decrease in the size of the aortic annulus (a 25 mm sized bio-prosthesis implanted in a 27 mm native annulus) caused the traction through the aorto-mitral continuity on the basal part of the anterior leaflet of the native mitral valve. Elimination of this traction by explanting the original bio-prosthesis and replacing it by self-expandable suture-less valve has solved the problem by restoring the original anatomical relationship between the aortic annulus and the anterior mitral valve leaflet (aorto-mitral continuity).

We cannot exclude some reduction of the afterload with the suture-less aortic bio-prosthesis that could contribute to a reduction of functional MR. However, the patient did not have any mitral valve disorders before the first AVR procedure, bot the first and redo (sutured) AVR were associated with good function and therefore we would not expect a significant reduction of the afterload by replacement of the sutured aortic bio-prosthesis by the suture-less aortic bio-prosthesis.

According to our case report the Perceval S suture-less bio-prosthesis was an effective rescue choice compared to standard sutured bio-prosthesis, which allowed avoiding significant deformities of the aortic annulus and aorto-mitral continuity with consequent mitral regurgitation.

## Conclusion

Aortic valve replacement using suture-less prosthesis could be a valuable optional choice for lowering the risk of deformation of the aortic annulus and aorto-mitral continuity. It might provide better outcomes in combined procedures with mild mitral regurgitation without indication for mitral surgery and avoid the possible iatrogenic lesion in such cases.

Further studies are needed to investigate the relationship between suture-less prosthesis and the adjacent anatomical structures of the aorto-mitral continuity, aortic root and annulus. Also effects on the mitral valve function (with or without surgical Intervention in the mitral valve itself) deserve further attention.

## Data Availability

The data set supporting the conclusions of this article is included within the article, and any other inquiry is available from the corresponding author on reasonable request.

## Abbreviations

TAVI: Transcatheter aortic valve implantation
AVR: Aortic valve replacement
SU AVR: Surgical aortic valve replacement
mmHg: Millimeter of mercury
A-M: Aorto-mitral continuity
XL: X large
AVR: Aortic valve replacement
