# Correction: Severe mitral valve insufciency caused by standard surgical aortic valve implantation and its reparation using sutureless prosthesis

**DOI:** 10.1186/s13019-023-02469-x

**Published:** 2023-12-08

**Authors:** Mahmoud Al‑Obeidallah, Marián Kohut, Milan Štengl

**Affiliations:** 1 Cardiac surgery department of Faculty Hospital in Pilsen, Charles University, Prague, Alej Svobody 80, Pilsen, 304 60 Czech Republic; 2 Department of Physiology, Faculty of Medicine in Pilsen, Charles University, Svobody, 1655/76, Pilsen, 323 00 Alej Czech Republic

**Correction to: Journal of Cardiothoracic Surgery (2023) 17:1**

10.1186/s13019-022-01896-6

Following publication of the original article [1], the given and family names of Marián Kohut were incorrectly structured. The name was displayed correctly in all versions at the time of publication.

The original article has been corrected.

